# Optimizing the Recovery of Rare Earth Elements from Spent Fluorescent Lamps by Living *Ulva* sp

**DOI:** 10.1021/acssusresmgt.4c00104

**Published:** 2024-07-11

**Authors:** Thainara Viana, João Colónia, Daniela S. Tavares, João Pinto, Nicole Ferreira, Jéssica Jacinto, Eduarda Pereira, Bruno Henriques

**Affiliations:** † LAQV-REQUIMTE − Associated Laboratory for Green Chemistry, Department of Chemistry, University of Aveiro, 3810-193 Aveiro, Portugal; ‡ CICECO − Aveiro Institute of Materials, Department of Chemistry, University of Aveiro, 3810-193 Aveiro, Portugal

**Keywords:** e-waste, valorization, rare earth elements, *Ulva* sp, biosorption

## Abstract

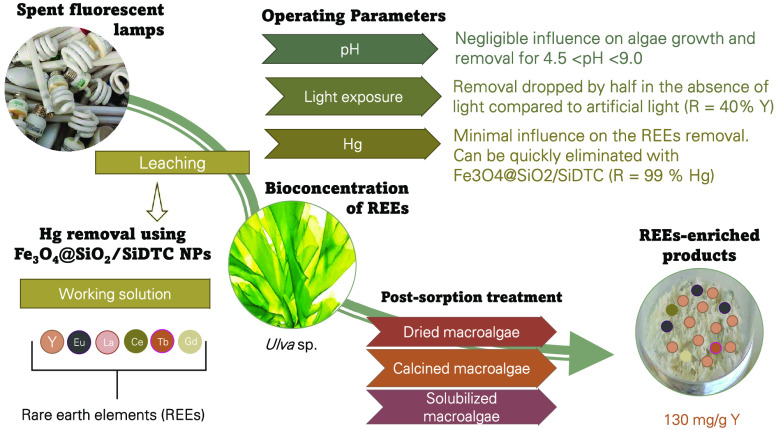

Given the significant industrial applications of rare earth elements (REEs), supply chain constraints, and negative environmental impacts associated with their extraction, finding alternative sources has become a critical challenge. Previously, we highlighted the potential of living *Ulva* sp. in the removal and pre-concentration of Y from a solution obtained by sequential acid leaching of spent fluorescent lamps (SFLs). Here, we extended that study to other REEs extracted from SFLs and evaluated the effect of pH (4.5–9.0), light exposure (absence, natural and supplemented with artificial light), and Hg (presence and absence). The results showed small differences in the removal of Y (23–30%) and other REEs at the different pH values, opening the scope of the methodology. However, *Ulva* sp. relative growth rate (RGR) was negatively affected in the higher acidity condition, without any visible signs of decay. In the absence of light, the RGR also decreased, which was accompanied by a halving of the removal efficiency compared to that with artificial light supplementation (40% for Y). Although Hg had minimal influence on the removal and concentration of REEs by *Ulva* sp., its presence in the enriched biomass is undesirable. Therefore, this contaminant was selectively removed from the solution using Fe_3_O_4_@SiO_2_/SiDTC nanoparticles before contact with the macroalgae (70% removal in 30 min; 99% in 72 h). In addition to easy solubilization, macroalgae enriched with REEs have a simpler composition compared to SFLs. Calcination of the biomass allowed the REEs to be further concentrated, with concentrations (130 mg/g for Y) up to 240 times higher than in typical apatite ore. This highlights enriched biomass as a sustainable alternative to traditional mining for obtaining these critical raw materials.

## Abstract 2

Given the industrial importance of rare earth elements, supply chain constraints, and environmental impacts, exploring alternatives like *Ulva* sp. for REE recovery from spent fluorescent lamps is promising despite challenges related to pH, light and mercury.

## 1 Introduction

From traditional industries (*e.g.*, agriculture, metallurgy) and general applications (*e.g.*, catalysts, polishes) to innovative and sustainable technologies (*e.g.*, phosphors and diodes in energy-saving lighting, permanent magnets in wind turbines), rare earth elements (REEs) have a wide range of applications.^1^ Due to ever-growing demand and supply challenges, these 17 elements of the periodic table are listed as Critical Raw Materials by Europe and the United States.^2,3^ Obtaining REEs from sustainable secondary sources as an alternative to environmentally harmful mining (primary source) is increasingly seen as the only way forward.^4,5^ Waste electrical and electronic equipment (commonly referred to as *e*-waste) has high potential as a secondary source because it is increasingly abundant and contains a composition richer in REEs than that of ores.^6^ In 2019, 54 Mt of *e*-waste were generated with a raw material value of $57 billion.^7^ Spent fluorescent lamps (SFLs) contain around 25,000 tons of REEs worldwide,^8^ including yttrium (Y), europium (Eu), gadolinium (Gd), lanthanum (La), terbium (Tb), and cerium (Ce).^9^ The Y content in SFLs varies between 30 and 35%, which is much higher than that in natural deposits (monazite ≈5%, bastnaesite less than 1%, clays less than 1%).^10^ Nevertheless, the recycling and recovery rates of REEs from SFLs and other e-waste are still low, partly because the REEs recovery processes are not yet economical and environmentally friendly.^11^

Most studies on obtaining REEs from SFLs focus on Y and Eu from red phosphor (YOX) oxides,^10,12^ and involve leaching the elements with concentrated acid followed by multiple precipitation/solvent extraction stages to concentrate and separate the REEs.^12−14^ Mechanical activation (ball milling) of SFLs can also be applied to facilitate the extraction of REEs from complex phosphor phases.^15^ Nevertheless, these methods have disadvantages in terms of high acid input, high energy input and/or expensive solvents, and are becoming increasingly unattractive given the generation of toxic waste.^15,16^ To minimize these setbacks, alternative methods using greener solvents or lower solvent concentrations have been proposed. For example, in a previous work, we successfully managed to extract Y from SFLs using a two-phase extraction method with less concentrated HNO_3_ (0.5 and 2 M), which also reduced the presence of undesirable elements such as Ca in the extract, leaving 99% of the Hg in solid waste.^11^

Due to their cost-effectiveness, affordability, and simplicity, a variety of biosorbents have been investigated for the preconcentration and recovery of REEs from aqueous solutions.^17^ Bacteria,^18,19^ mosses,^20^ fungi,^21^ yeasts,^22^ and photosynthetic organisms such as micro-^23^ and macroalgae^24−26^ have been explored. Compared to microalgae or non-living biomass, living macroalgae have several advantages: no pre-treatment is required, which reduces costs;^27,28^ easier separation from solution;^29^ potentially higher efficiency due to intracellular accumulation and continuous biomass growth;^29^ and CO_2_ capture, reducing carbon footprint.^30^

Marine macroalgae, particularly those belonging to the *Ulva* genus, have been target for REE extraction from synthetic mono- and multielement solutions with superior performance over other species^31,32^ due to their intrinsic properties such as high specific surface area and cell wall sulfate polysaccharide Ulvan (from *Ulva* sp., with high affinity for REEs),^33^ and ability to adapt to a wide salinity range.^34^ Recently, we transposed it into a real context, demonstrating, for the first time, the potential of *Ulva* sp. in the removal and pre-concentration of Y from a solution obtained from the sequential acid leaching of a real SFL residue.^11^ Some important operational parameters (sorbent dosage, salinity, and initial Y concentration) were studied and optimized through the Response Surface Methodology.^11^

The present work arose intending to evaluate other relevant operational parameters, which can impair or improve the performance of the living macroalgae, such as the pH of the medium and supplementation with artificial light, in addition to extending the previous study^11^ to other REEs extracted from SFLs (Eu, Gd, and Tb). Along with REEs from the SFLs, some Hg is also leached, and its presence is undesirable. The mercury problem is of great importance in the used lamp recycling sector. Due to its toxic effects, the applied methodology may be hindered. Therefore, this priority contaminant was removed from the leachate using Fe_3_O_4_@SiO_2_–SiDTC nanoparticles developed by the research team. The performance of macroalgae in removing and preconcentrating REEs from the leachate in the absence and presence of Hg was then evaluated. Finally, to obtain the highest amount of REEs per gram of macroalgal biomass, post-sorption calcination of macroalgal biomass was investigated. The composition of the REE-enriched dried biomass, the REE-enriched calcined biomass and the aqueous solution resulting from the solubilization of the REE-enriched dried biomass were characterized to evaluate potential “final products”.

## 2 Materials and Methods

### 2.1 Reagents and Materials

All chemical reagents used are classified as analytical grade and were obtained from certified suppliers. Stock standard solutions for the calibration of analytical equipment were obtained from Inorganic Ventures (certified reference materials for Inductively coupled plasma). Mercury (Hg) stock solution was obtained from Merck. Nitric acid (HNO_3_, 65% m/m), hydrochloric acid (HCl, 30% m/m), and ultrapure Milli-Q water (18 MΩ/cm) were used in the preparation of the calibration standards and throughout the extractions.

All the material used was previously washed with Milli-Q water, immersed in a solution of HNO_3_ 25% (v/v) for at least 24 h and rinsed with Milli-Q water before use. Glass vessels used in the storage of solutions for Hg analysis were additionally immersed in a solution of HNO_3_ 65% for at least 48 h before reuse.

### 2.2 Waste Preparation

The waste resulting from the dismantling and treatment of spent fluorescent lamps (SFLs) was kindly supplied by a Portuguese recycling company specializing in this type of waste. The sludge was dried, homogenized and sieved to Ø < 0.2 mm (to eliminate the presence of glass, plastic, or metallic filaments) according to Pinto et al.^11^

### 2.3 Waste Characterization

Chemical characterization was performed through inductively coupled plasma optical emission spectrometry (ICP-OES) on a Horiba Jobin Yvon Activa M. Quantified elements were: yttrium (Y), lanthanum (La), cerium (Ce), europium (Eu), gadolinium (Gd), terbium (Tb), aluminum (Al), manganese (Mn), iron (Fe), nickel (Ni), copper (Cu), zinc (Zn), lead (Pb), boron (B), sodium (Na), magnesium (Mg), calcium (Ca), and potassium (K). A microwave-assisted acid digestion was applied to the waste before quantification, following the procedure described in Pinto et al.^11^

Mercury was also quantified, by cold vapor atomic fluorescence spectroscopy (CV-AFS) on a PSA cold vapor generator (model 10.003) connected to a Merlin PSA detector. As the reducing agent, tin chloride (SnCl_2,_ 2% w/v in 10% v/v HCl) was used. Quality control was ensured by analyzing each sample in at least triplicate, accepting results with a coefficient of variation of ≤5%.

### 2.4 Saline Water and *Ulva* sp. Collection

Saline water was collected from the Atlantic Ocean, Aveiro, Portugal (salinity 35; 40°64′41′′N, 8°74′53′′W) during high tide. Opaque containers were used for transportation to block light and prevent possible changes in properties (*e.g.*, microalgae proliferation). The water characterization in terms of pH value, conductivity as well as main and secondary ions corresponded to that of Henriques et al.^35^ The saline water was filtered with Millipore porous filters (0.45 μm) and diluted to a salinity of 10 with ultrapure water (18 MΩ/cm), for later use.

The marine macroalgae *Ulva* sp. was collected in the Ria de Aveiro lagoon on the northwest coast of Portugal (40°38′N, 8°45′W). This cosmopolitan species is tolerant to fluctuations in salinity that occur naturally in its habitat due to the inflow and outflow of seawater during high and low tides. In the laboratory, the macroalgae were washed to remove sediment and organisms that could interfere with the sorption process. *Ulva* sp. was kept in aerated aquariums filled with saltwater of the desired salinity, under natural sunlight (photoperiod of 12/12 h) and at room temperature (20 ± 2 °C). A portion of the sampled macroalgae was stored at −80 °C and freeze-dried to quantify the basal concentrations of REEs in the pristine seaweed tissue.

### 2.5 Biosorption Experiments

To obtain the working solution, which contains the REEs and other elements, a two-step extraction methodology was applied to the dry SFL waste. This previously optimized methodology^11^ allows us to obtain a solution rich in Y and Eu, while minimizing the concentrations of the remaining (undesirable) elements. The composition of the extract/leachate matched that of Pinto et al.^11^ The working solution contained approximately 120 mg/L Y, 7.0 mg/L Eu, 0.32 mg/L Gd, 0.098 mg/L Tb. Cerium and lanthanum were below the limit of quantification (10 μg/L).

The performance of *Ulva* sp. on the removal and concentration of REEs from the working solution under different pH conditions, light exposure and presence/absence of Hg was evaluated. Assays were conducted in 1 L Schott Duran flasks, at room temperature (20 ± 2 °C), salinity 10 and sorbent dosage (9 g/L), conditions optimized in previous work.^11^ Assays were performed in triplicate.

To minimize intra-assay differences and evaluate relative growth rate (RGR,%/day) along exposure, *Ulva* sp. were cut into discs (Ø = 3.5 ± 0.1 cm). Saline water was diluted with Milli-Q water to salinity 10 (measured with an Eclipse model 45–63 handheld refractometer). Control solutions (working solution without *Ulva* sp.) and blank solutions (clean saline water with *Ulva* sp.) solutions were also analyzed. Water sampling was carried out immediately before the addition of the seaweed (0 h) and after 1, 3, 6, 9, 24, and 48 h of contact. Samples were acidified with 65% HNO_3_ and stored at 4 °C until quantification. Macroalgae samples were stored at −80 °C.

#### 2.5.1 Study of the Influence of pH

The influence of pH on the removal/pre-concentration of REEs by *Ulva* sp. was evaluated by conducting assays at pH 4.5; 6.0; 7.5; and 9.0, in a similar manner as described previously. In addition to determining the pH value at which the sorption process is most efficient, it was also aimed to assess the pH range tolerated by *Ulva* sp. without sorption impairment.

#### 2.5.2 Study of the Influence of Light Exposure

Since the proposed process is based on a photosynthetic organism, it is important to examine the influence of light exposure on the removal/preconcentration of REEs by *Ulva* sp. In the present work, three different exposure conditions were carried out: natural light (approx. 12 h L: 12 h D); natural light continuously supplemented by artificial light (LED growth lamp with wavelengths of 640–680 nm and 440–480 nm, 36 W) (24 h L); and complete absence of light (24 h D). The assays followed the procedure described previously.

#### 2.5.3 Study of the Influence of Mercury Presence

Although the two-step extraction methodology minimizes the solubilization of Hg from the SFL waste (99% remains in the residue), the leachate still contains a relatively high concentration of this top-priority contaminant (≈ 600 μg/L). The removal of Hg is relevant, particularly if it: a) affects the REEs removal process by *Ulva* sp. or b) is incorporated by the organism, being present in the “final product”.

In a previous work, Fe_3_O_4_@SiO_2_–SiDTC nanoparticles showed a high ability to remove Hg from different water matrices, at realist environmental concentrations.^36^ In the present work, the removal of Hg from the leachate was evaluated, in polypropylene flasks (1 L) using 50 mg/L of Fe_3_O_4_@SiO_2_–SiDTC nanoparticles under continuous stirring. Sampling was performed immediately before the addition of the sorbent (0 h) and after 0.5; 2; 6; 24; 48 and 72 h. All samples were acidified with 65% HNO_3_ and stored at 4 °C until quantification.

The influence of Hg on REEs removal/pre-concentration by *Ulva* sp. was then evaluated by conducting assays in the working solution with and without pre-treatment with Fe_3_O_4_@SiO_2_–SiDTC nanoparticles. Assays followed the procedure described previously, at optimal pH and light exposure conditions (defined from 2.5.1 and 2.5.2).

### 2.6 Calcination and Chemical Composition of Macroalgal Biomass Post Biosorption

After sorption experiments, the macroalgae were briefly placed on absorbent paper to remove excess water on the surface, then placed on aluminum foil, dried in an oven at 30 °C for 48 h until a constant weight was reached, and analyzed.

A part of the macroalgae was calcined to pre-concentrate the sorbed REEs. Approximately 1 g of dried biomass, previously homogenized by grinding, was placed in a porcelain crucible at 900 °C (raising temperature rate of 25 °C/min) for 1 h.^37^ The calcined algae were collected and digested for further element quantification.

The solubilization of the macroalgal biomass post-sorption was done by microwave-assisted acid digestion, following the methodology described in Jacinto et al.^31^ Approximately 200 mg of the sample was weighed into Teflon vessels and digested in a CEM MARS 5 microwave, model 240/50, with continuous monitoring of temperature and pressure. The digestion solutions were collected in 25 mL polyethylene bottles and the volume was filled with ultrapure water. The quality control of the method was ensured by parallel digestion of procedure blanks (reaction vessels with reagents and without sample), which were always below the limit of quantification, and certified reference material (NIST SRM 1515 - apple leaves), whose recovery was always in the range of 84–100%.

### 2.7 Formulas and Statistics

The relative growth rate (RGR, %/day) of *Ulva* sp. during the experiments was calculated by measuring the initial (*A*_0_) and final (*A*_*t*_) areas of the discs (Ø = 3.5 ± 0.1 cm), assuming an exponential growth model,^25^ where *t* is the time in days:
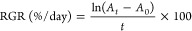


The amount of REEs removed from the working solution (Removal, %) was assessed based on the initial (*C*_0_) and final (*C*_t_) concentrations of REEs in the solution, following the equation:
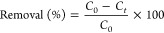


Assuming that all REEs removed from the solution were biosorbed/bioaccumulated by *Ulva* sp., the expected concentration of REEs in the macroalgal biomass at the end of experiments (*q*_*t*,calculated_, μg/g) was estimated by
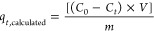
where *V* (L) is the volume of solution and *m* (g, dry weight) is the macroalgae mass.

The actual concentration of REEs in *Ulva* sp. at the end of the experiments (*q*_*t*_,_observed_, μg/g) was calculated by the difference between the final (*q*_*t*_, μg/g) and baseline (*q*_0_, μg/g) concentrations in the biomass, determined by ICP-MS following microwave-assisted acid digestion:
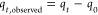


## 3 Results

### 3.1 Influence of pH

**Figure 1:**
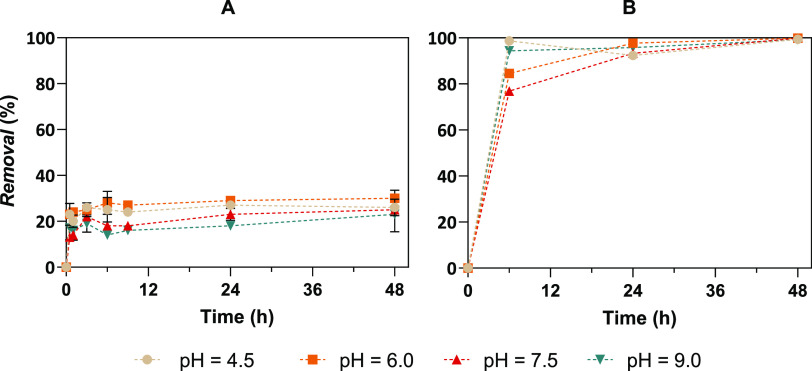
Removal (%) of: A, Yttrium and B, Mercury from the working solution along time (*t*, h) for the different initial pH studied (4.5 (●), 6.0 (■), 7.5 (▲) and 9.0 (▼)) in the presence of *Ulva* sp. Results are expressed as mean ± standard deviation (*n* = 3).

At all pH values (4.5; 6.0; 7.5; 9.0), the removal of Y from the working solution, after 48 h of contact with *Ulva* sp., was greater than 23% (Figure 1A), with the highest removal found at pH 6.0. The observed kinetic profiles were almost coincident and showed fast kinetics for Y removal (pseudo-equilibrium was reached at *t* = 6 h for pH 4.5 and 6.0, with a slight increase in the remaining times analyzed). Europium removal varied between 17% (pH 7.5) and 29% (pH 4.5). For Hg (Figure 1B), the removals at *t* = 6 h were higher than 75% at all pH studied and reached more than 90% at pH 4.5 and pH 9.0. At the end of exposure, Hg removal was ≥99% under all conditions examined. Overall, none of the evaluated pH values had a negative impact on the removal of elements from the working solution. The lowest residual concentrations of Y in the solution reached ≈84 mg/L. In the absence of *Ulva* sp., the concentration of the elements remained relatively stable at all pH values studied (data not shown).

**Figure 2:**
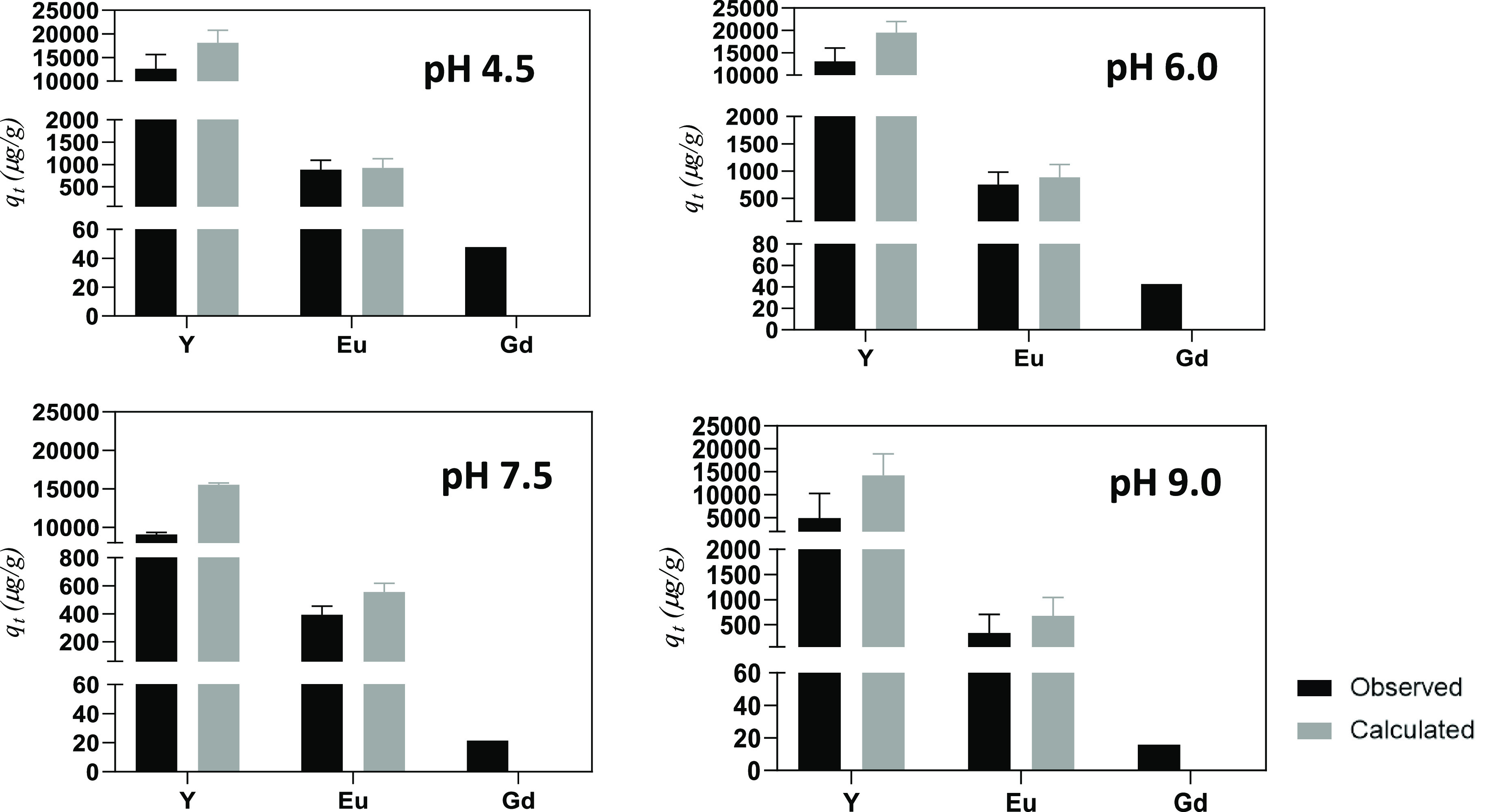
Amount of REEs per mass of *Ulva* sp. (*q*_*t*_, μg/g) calculated from mass balance (grey) and obtained from the quantification by ICP-OES (black) at different initial pH values (4.5; 6.0; 7.5; 9.0) of the diluted working solution.

The contents of REEs in *Ulva* sp. before and after contact with the working solution are summarized in Figure 2. The initial concentrations in the macroalgae biomass were below the limit of quantification (10 μg/g for Y and 5.2 μg/g for the remaining REEs), which was also observed for macroalgae from the blank (data not shown). Macroalgae exposed to the working solution at different initial pH values revealed a significant increase in REEs concentration: 9–15 mg/g for Y, 0.4–0.9 mg/g for Eu and 16–48 μg/g for Gd. The differences between the *q*_*t*_ values obtained by quantification and the values calculated by mass balance were relatively small, with the largest differences detected at pH 7.5 and 9.0 (Figure 2).

Regarding the physiological status of *Ulva* sp., the relative growth rate (RGR,%/day) after 48 h for the macroalgae in the blank varied between 4.7 and 6.8% (Table S1, supplementary material). An increasing profile as a function of pH was observed in the macroalgae exposed to the working solutions, with the RGR varying between 1.1 and 6.8%. The chlorophyll content slightly decreased when comparing the values of the macroalgae in the blank condition (12–16 SPAD units) and the macroalgae exposed to the working solution (11–13 SPAD units).

The pH was monitored *ex-situ* during the 48-h exposure (Figure S1, Supplementary Material). A large fluctuation in the pH value was observed in the blank. The pH of the exposure conditions showed a slight increase at *t* = 6 h, followed by a decrease, tending to a pseudo-equilibrium state, like the control conditions variation (decreased in the first 6 h and remained constant until the end of exposure).

### 3.2 Influence of Light Exposure

**Figure 3:**
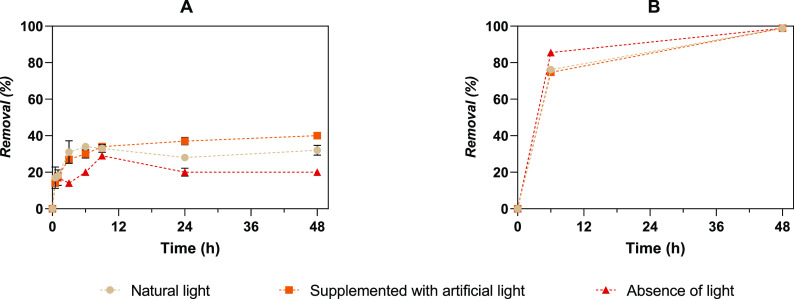
Removal (%) of: A, Yttrium and B, Mercury from the diluted working solution along time (*t*, h) for the different light exposures studied (natural light (●), natural light supplemented with artificial light (■) and absence of light (▲)) in the presence of *Ulva* sp. Results are expressed as mean ± standard deviation (*n* = 2).

The results showed that the absence of light is reflected in slower kinetics and fewer REEs removed from the solution (20%, 15%, 18%, and 12% for Y, Eu, Gd and Tb, respectively) (Figure 3A). The removal of Y from the working solution when supplemented with artificial light reached a removal of 40% for Y, which is higher than that observed with exposure to natural light (30%). For the remaining REEs, maximum and minimum removals were achieved with the supplementation and absence of light, respectively, corresponding to 15 and 23% for Eu, 18 and 32% for Gd, and 13 and 34% for Tb. The removal kinetics of Hg (Figure 3B) showed no negative influence when different light exposures were assessed. At time *t* = 6 h, more than 75% of Hg was removed, reaching ≥99% at the end of the exposure, in all studied conditions.

**Figure 4:**
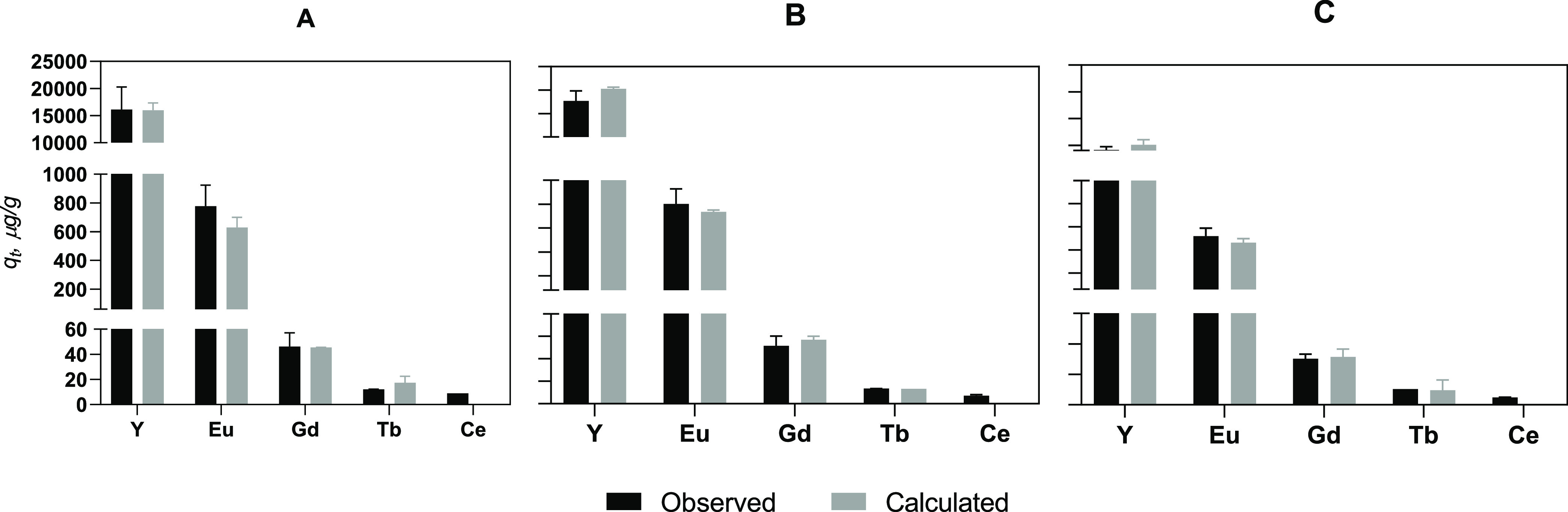
Amount of REEs per mass of *Ulva* sp. (qt, μg/g) calculated from mass balance (grey) and obtained from the quantification by ICP-OES (black) at different light exposures (A, natural light; B, supplemented natural light; C, absence of light) of the diluted working solution.

Figure 4 shows a good agreement between predicted and observed concentrations of REEs (*q*_*t*_, μg/g) in *Ulva* sp. biomass after exposure to the working solution under different light exposure conditions. The Y content in the macroalgae varied between 9 and 18 mg/g, with the highest values achieved under the condition supplemented with artificial light. The remaining REEs concentrations varied between 0.5 and 0.8 mg/g for Eu, 30 to 52 μg/g for Gd, 10 to 14 μg/g for Tb, and 5 to 9 μg/g for Ce.

The RGR of the macroalgae exposed to the working solution varied between 5.2 and 6.5%/day after 48 h (Table S2, Supplementary Material). For the macroalgae in the extract-free treatment (Blank), the RGR varied between 7.4 and 9.8%/day. The chlorophyll content decreased considerably when comparing the values for macroalgae in the blank (12–15) and macroalgae exposed to the working solution (9.6–10).

Variation in pH showed an increase in alkalinity under extract-free conditions, except for no-light condition, where an increase in acidity was observed. The pH variation for working solutions and corresponding control solutions was similar to that observed when different initial pH were evaluated (Figure S2, Supplementary Material).

### 3.3 Influence of Mercury Presence

**Figure 5:**
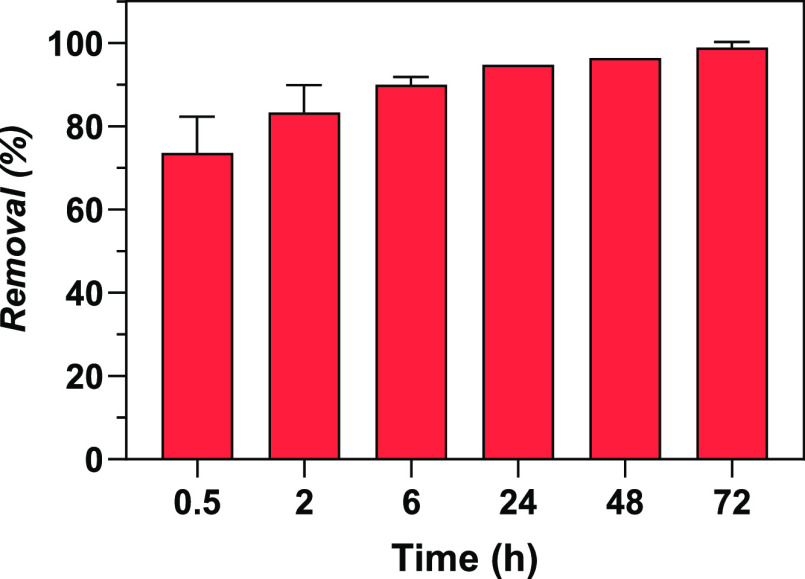
Removal (%) of Hg from extract/leachate during contact time (h) with 50 mg/L Fe_3_O_4_@SiO_2_–SiDTC. Initial concentration of Hg ≈600 μg/L; pH = 0.1. Results are expressed as mean ± standard deviation (*n* = 2).

Figure 5 shows the time evolution of Hg removal from the extract/leachate by the Fe_3_O_4_@SiO_2_–SiDTC nanoparticles. Despite the extremely low pH (0.1), the removal efficiency was over 70% after 30 min of contact and increased progressively over time, reaching about 99% after 72 h. After 24 h, the residual concentration of Hg in the solution was 31 μg/L (Hg values below the legal limit) and reached 7 μg/L, after 72 h.

**Figure 6:**
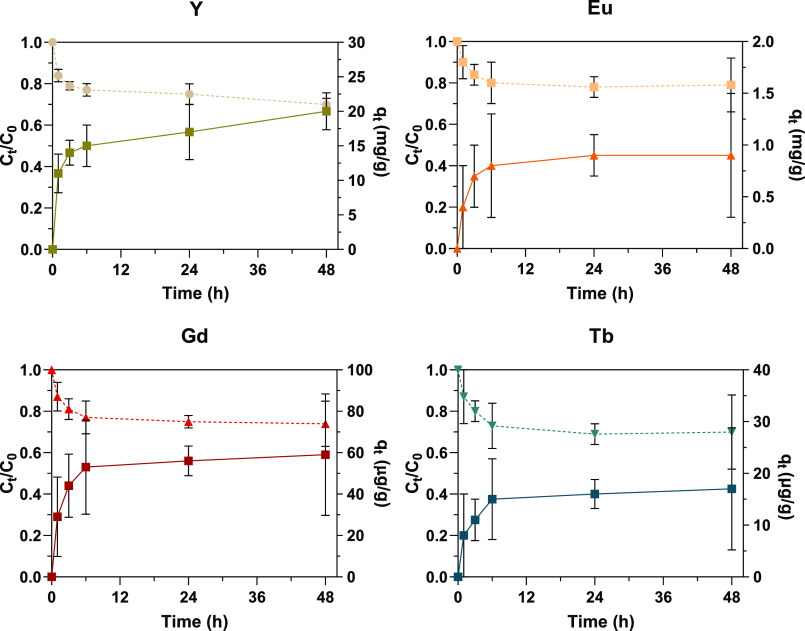
Ratios between concentrations of Y, Eu, Gd, and Tb at time *t* (*C*_t_) and at initial conditions (*C*_0_) in the in the Hg-free working solution (dashed line) and calculated concentrations of REEs in *Ulva* sp. biomass (qt; continuous line) along time. Results are expressed as mean ± standard deviation (*n* = 3). Experimental conditions: 9 g/L of *Ulva* sp., Initial concentration of Y of 120 mg/L, pH 6.0, salinity 10.

Figure 6 shows the kinetics of the removal of Y and other REEs from the working solution by *Ulva* sp., after Hg was removed with Fe_3_O_4_@SiO_2_–SiDTC nanoparticles. The removal profiles were like those observed in the presence of Hg (maximum removals of 30, 21, 26, and 30% for Y, Eu, Gd and Tb, respectively). The concentration of REEs in *Ulva* sp. biomass (*q*_*t*_) also followed values previously obtained in the presence of Hg (20 and 0.9 mg/g for Y and Eu, respectively and 59 and 17 μg/g for Gd and Tb, respectively).

### 3.4 Postsorption Concentration of REEs

**Figure 7:**
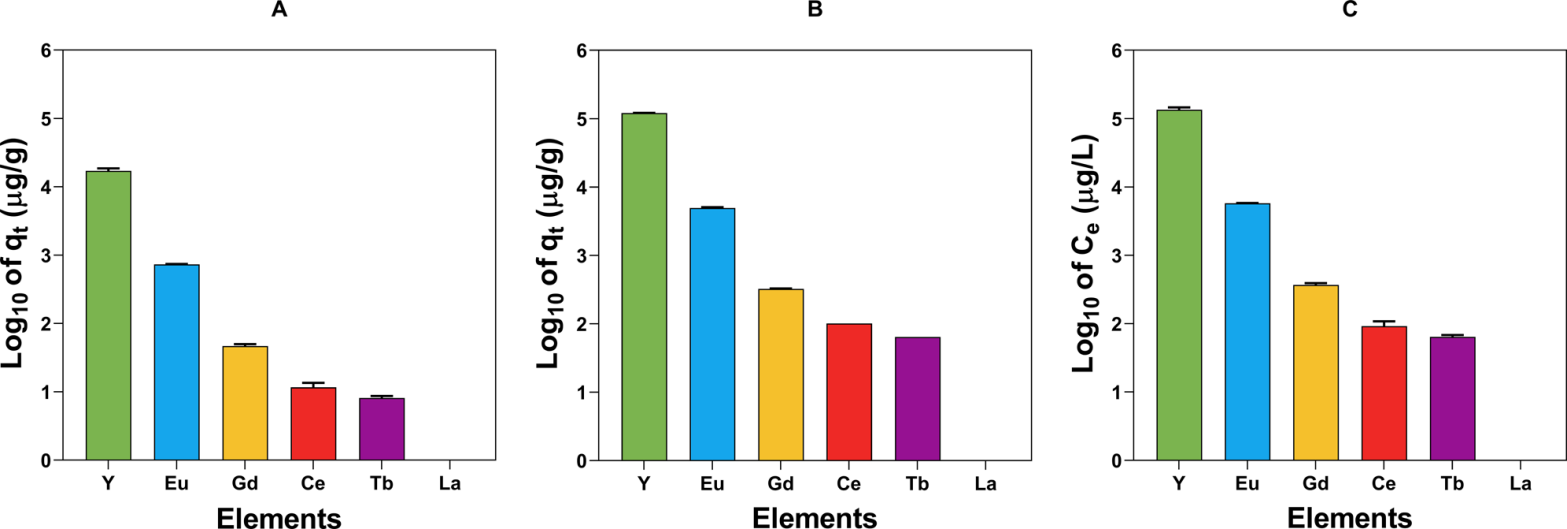
Logarithmic concentration of REEs in *Ulva* sp. (*q*_*t*,observed_) for: A, dried biomass; B, calcinated biomass; C, solubilized dried biomass.

Figure 7 shows the concentration of Y and other REEs in the dried and calcinated algal biomass. The results showed a high ability of macroalgal biomass to preconcentrate Y, which increases with the calcination process (16.4 and 121 mg Y per gram of *Ulva* sp., pre- and post-calcination, respectively). Other REEs were also pre-concentrated in the algal biomass (*e.g.*, ≈5.0 and 0.7 mg/g Eu in the calcinated and dried biomass, respectively). By solubilizing the dried macroalgae after sorption, a solution with a high Y concentration (up to 132 mg/L) could be obtained (Figure 7). The concentrations of REEs and elements can be seen in Tables S3 and S4, Supplementary Material.

**Figure 8:**
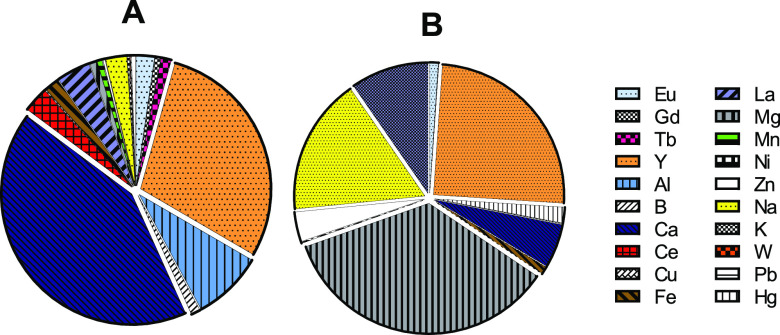
Relative element composition in A, starting residue; B, calcined biomass; considering the elements that were quantified.

Comparing the calcined macroalgae with the starting residue (Figure 8) it was found that the former has a simpler composition with fewer non-interest elements. In addition to a higher mass concentration of Y (121 mg/g versus 91 mg/g) and no Hg, calcined macroalgae are essentially composed of Mg, Na, K and to a much lesser extent, Ca. Please note that Figure 8 only presents the relative percentages of the elements that were quantified and not total concentrations.

## 4 Discussion

The wide range of applications for REEs is directly reflected in their high demand. The recycling of REEs from secondary sources, *e.g.*, *e*-waste, is considered extremely important to reduce the exploitation of primary sources and reduce the associated negative environmental impacts. Not only is the concentration of REEs in the SFLs of considerable interest, but also a more environmentally friendly biotechnological approach to recover these elements from this waste has been highlighted by our research group.^11^ Several parameters can overall influence the sorption performance of the biosorbent and have been extensively discussed in the literature.^38,39^ An important factor that may impair the sorbent performance is the pH of the medium. This factor influences both the chemistry of metal ions and the chemistry of the functional groups of biosorbents.^40^ When using a living organism, pH can also affect metabolic functions, photosynthesis and physiological aspects such as growth rate, which can influence sorption efficiency.^41^ An important result of the present work was the observation that the kinetic removal profiles were briefly influenced by pH (from 4.5 to 9.0), indicating a relatively wide range of applications and that incidental fluctuations in the pH will not severely affect the efficiency of the process. Nevertheless, the REEs concentration in *Ulva* sp. and the calculated BCF decreased at pH 9, which could mean that not all REEs removed from the solution were due to the biosorption capacity of the macroalgae. The geochemical behavior of REEs in the aquatic environment is strongly pH-dependent and speciation shifts regulate the solubility and bioavailability of these elements.^42,43^ In chloride media, REEs precipitation can occur at pH > 6.8–8.0, and a shift in the pH of the precipitation can occur when the medium is changed (*e.g.*, in sulfide medium precipitation occurs at lower pH values and in nitrate medium insoluble complexes are formed at slightly higher pH values compared to chloride).^44,45^ With a view of a circular economy and the reintroduction of REEs into the production cycle, a higher concentration of these elements in the algal biomass is preferable, minimizing process losses.

When in contact with the working solution, the RGR of the macroalgae was only slightly reduced compared to the extract-free condition (blank), an effect that was minimized with the increase in pH, and negligible alterations in the total chlorophyll content were observed. Exposure to high metal concentrations or a myriad of different contaminants has been shown to negatively affect and even suppress some physiological parameters of algae (chlorophyll content, growth, photosynthesis, polysaccharide content and structure).^46,47^ In this sense, the choice of *Ulva* species was crucial since its ability to tolerate the presence of different contaminants has been well described in the literature. A proper growth rate is also an important aspect of the process since it is directly related to the sorption efficiency because biomass increase leads to more surface area and new binding sites.^46,48^ Green seaweed *Ulva* are known to have rapid growth, almost equivalent to that of microalgae^49^ and can be combined with other processes (*e.g.*, biorefinery) and act as a CO_2_ sink (which stimulates *Ulva* blooms).^50,51^

As an ecological factor, light exposure influences the growth of photosynthetic organisms. Some studies even suggest that the growth of *Ulva* is more influenced by light rather than by the inorganic carbon source.^51^ Light in excess causes synthesis of reactive oxygen species (ROS), or even is dissipated rather than contributing to biomass accumulation, which affect biomass yield and damage the photosynthetic machinery.^52^ Nevertheless, at high light intensity, low pH can trigger photoprotective processes and reduce photosynthesis and growth rate.^53^ This was slightly observed herein this study regarding algae growth with and without extract exposure even though higher REEs removal was achieved. In the complete absence of light, a decrease in RGR was also observed, accompanied by a halving of the removal efficiency compared to artificial light supplementation. This may be attributed to a stress combination of lack of irradiance and a more acidic medium.^54^ Photosynthetic organisms have evolved two primary ways of assimilating inorganic carbon: photosynthesis and respiration.^55^ The respiration process occurs in reduced/absent irradiation and involves oxygen consumption and CO_2_ production, which lowers the pH of the solution^30^ as it was seen in the extract-free condition. The respiration rate is important as it significantly decreases the light-to-biomass conversion,^56,57^ consequently reducing the availability of binding sites for REE and lessen the removal efficiency. Although the rate of respiration at night is also modulated by irradiance levels experienced during the day, in this study no irradiance was performed, thus depletion of carbohydrate reserves over time could have occurred. The stress responses could include alterations in metabolic pathways, and adjustments in physiological processes to cope with the unfavorable conditions (*e.g.*, *S. latissima* tends to reduce the consumption of carbohydrates to save energy under prolonged darkness and inhibited the biosynthesis of cell wall polysaccharides).^58^ Although the addition of artificial light has resulted in higher REEs removal efficiency, a positive point of the present work is the fact that natural light by itself allows a removal that is not significantly lower and may be advisable from the point of view of reducing electrical energy consumption.

Mercury is considered a problem in the recycling sector of spent lamps. According to the European Directive on the Restriction of Hazardous Substances in Electrical and Electronic Equipment (RoHS) and further amendments, a maximum Hg content of 2.5 mg per lamp is permitted.^59,60^ In lamps, Hg is distributed between powder (12.1%), end-cap (12.9%), glass (8.3%), and vapor (67%).^61^ The methodology described in the present work allows the extraction of Y and Eu while retaining 99% of the Hg in the solid residue, and the application of Fe_3_O_4_@SiO_2_/SiDTC nanoparticles, under the tested experimental conditions, enabled the removal of the Hg that migrated during the REEs leaching step without interacting with REEs. The Hg concentration in the working solution decreased to nearly 7-fold below the old legal values, 50 μg/L.^62^ Removal is kinetically rapid (70% after 30 min), even taking into account the high acidity and ionic competition of the medium, and in comparison to previously reported work: Girginova et al.^63^ reported 74% of removal from ultrapure water after 48 h of contact ([Hg]_0_ = 50 μg/L, Fe_3_O_4_/SiO_2_/NH/CS_2_^–^ mass = 3 mg/L); Figueira et al.^64^ reported more than 98% of removal from saline water after 96 h of contact ([Hg]_0_ = 50 μg/L, Fe_3_O_4_/SiO_2_/NH/CS_2_^–^ mass = 6 mg/L); and Hakami et al.^65^ reported ≈100% of removal from ionic competition-free solution after 15 min of contact ([Hg]_0_ = 80 μg/L, Thiol-functionalized mesoporous silica-coated magnetite nanoparticles mass = 8 mg/L).

Although the efficiency of removal of REEs by macroalgae was not statistically different in the presence and absence of Hg, the presence of this contaminant in the REEs-enriched biomass must be avoided as it may affect subsequent purification processes, which is successfully achieved by the application of Fe_3_O_4_@SiO_2_/SiDTC. Magnetic nanoparticles (MNPs) are marked as promising materials for sorption mainly due to their capacity to overcome the drawbacks related to the application of conventional separation techniques.^66^ However, MNPs have not yet replaced conventional technologies in any field due to lack of toxicity and hazard analysis, challenges in cost-effective commercial synthesis and availability, limited understanding of the performance of different MNPs at varying conditions, and lack of studies on stabilization of MNPs for on-field usage.^67^

An alternative approach to eliminating Hg from the enriched biomass can be calcination of the biomass (in the present work, 98% of the Hg could be eliminated).^68,69^ However, the gases produced during calcination contain high concentrations of Hg and an industrial application necessarily requires a filtration system^70^ (*e.g.*, activated carbon-based sorbents), which must retain this element and prevent its release into the atmosphere to ensure regulatory compliance and environmental protection.

Rare earth elements are usually commercialized in the solid state as carbonates, oxalates, hexahydrate nitrates, oxides, or in its pure form.^71^ The purer the product is, the higher its market value. Leaching and pre-concentration processes are crucial for the quality (purity) and quantity of the final product.^71^ The simpler the composition of the matrix and the higher the relative concentration and availability of REEs in the matrix, the more efficient the purification process will be. In the present work, the combination of two-step leaching with subsequent biosorption by macroalgae allowed us to obtain biomass with a simpler composition and higher concentration in Y and Eu compared to the initial lamp residue. Furthermore, the solubilization of REEs in macroalgae biomass requires less aggressive means than those required for ore.^72,73^ Calcining the biomass increased the concentration of Y and other REEs up to 8 times and reduced the algal weight by 87%. This establishes the calcined algae (25% of Y in weight) as an alternative source of REEs, particularly Y, since the REEs content in natural deposits hardly surpasses 5% in weight.^74−76^ Compared to natural apatite ores, the obtained Y concentrations in dried and calcined algae were 32 to 240 times greater.^77^ The values found for the solubilized algae were higher than the initial concentration in the working solution and up to 8 times higher than the values found for ΣREEs in other secondary sources, *e.g.*, acid mine drainage (AMD)^78−80^ with less concentration of non-interest (interfering) elements, such as Fe and Ca. Findings herein obtained, when compared to other biosorption studies (Table S5), revealed that the proposed approach is one of a few to work with real FL waste. Without any surface modification, *Ulva* sp. was able to have a *q*_*t*_ for Y equal and/or superior to other sorbents without the calcination process (3–21 times superior after calcination). After calcination the *q*_*t*_ of Eu was in the same magnitude order as the max *q*_*t*_ of other sorbents. Although obtaining individualized elements was not the aim of the present work, the separation of Y from Eu can be further achieved by taking advantage of the differences in the oxidation states of the elements. Eu(III) can be reduced to Eu(II) and precipitated as sulfate, leaving Y in solution.^81^

## 5 Conclusion

The present study greatly expands the understanding of algae-based biotechnology as a viable option for e-waste treatment and recovery of rare earth elements. The results confirmed the viability of living *Ulva* sp. for recovery and concentration of Y and other REEs from SFLs leachate in a pH range of 4.5 to 9.0. Nevertheless, some differences in *q*_*t*_ (obtained vs. calculated) were observed at pH = 9, possibly due to precipitation as hydroxides/carbonates in solution. The effect of light exposure was found to be relevant, with the lowest and highest efficiencies of REE uptake by macroalgae recorded in the absence of light and under natural light supplemented by artificial light, respectively.

Although the presence of Hg in the solution had a negligible influence on the removal and concentration of Y or other REEs by the living *Ulva* sp., its presence in the enriched biomass is undesirable. By using Fe_3_O_4_@SiO_2_/SiDTC nanoparticles, this element could be quickly and selectively removed from the solution before contact with the macroalgae. At the end of the process, the enriched biomass has a simpler composition than the initial residue and is easily solubilized, which is beneficial for the final stage of purification/individual separation of elements. By calcining the macroalgae biomass, its volume can be considerably reduced and the REEs can be further concentrated. The concentration of Y in the calcined material is higher than that in the SFL residue and up to 240 times higher than that in ordinary apatite ore.

In this way, enriched biomass represents a sustainable alternative to mining to obtain these critical raw materials, which can be further processed using the implemented methods to purify the elements.

## Supporting Information Available

Relative growth rate (%/day), total chlorophyll and bioconcentration factor in the macroalgae exposed to saline water (B) and to the diluted extract (E) at different initial pH (Table S1) and variation of pH versus time of exposure at those conditions (Figure S1), relative growth rate (%/day), total chlorophyll and bioconcentration factor in the macroalgae exposed to saline water (B) and to the diluted extract (E) at different light exposure (Table S2) and variation of pH versus time of exposure at those conditions (Figure S2), concentration of REEs and other elements in *Ulva* sp. biomass (Tables S3–S4), literature review on the application of different adsorbents for REEs recovery (Table S5) (PDF)
