# Supplementary material for: Optimizing the Recovery of Rare Earth Elements from Spent Fluorescent Lamps by Living Ulva sp
Source: ACS Sustain Resour Manag. 2024 Jul 11;1(7):1464–74. doi: 10.1021/acssusresmgt.4c00104 (PMC11285805; doi:10.1021/acssusresmgt.4c00104)
Supplement: Supplementary file 1 — rm4c00104_si_001.pdf [file rm4c00104_si_001.pdf]

Supplementary material

Optimizing the recovery of rare earth elements from spent fluorescent lamps by living *Ulva* sp.

Thainara Viana[a], João Colónia[a], Daniela S. Tavares[a], João Pinto[a*], Nicole Ferreira[a,b], Jéssica Jacinto[a,], Eduarda Pereira[a], Bruno Henriques[a*]

[a] LAQV-REQUIMTE – Associated Laboratory for Green Chemistry, Department of Chemistry, University of Aveiro, 3810-193 Aveiro, Portugal

[b] CICECO – Aveiro Institute of Materials, Department of Chemistry, University of Aveiro, 3810-193 Aveiro, Portugal

*Corresponding authors: joao.pedro.pinto@ua.pt and brunogalinho@ua.pt

1.1. Results

***Table S1**: Relative growth rate (%/day), total chlorophyll and bioconcentration factor in the macroalgae exposed to saline water (B) and to the diluted extract (E) at different initial pH (4.5; 6.0; 7.5; 9.0). Chlorophyll analysis was performed with the SPAD-502 meter, thus the units of the analysis are proper of the device.*

	pH 4.5		pH 6.0		pH 7.5		pH 9.0	
	B	E	B	E	B	E	B	E
**RGR (%/day)**	4.7 ± 0.9	1.1 ± 0.2	6.8 ± 0.0	2.6 ± 0.3	5.2 ± 2.0	3.3 ± 0.4	4.8 ± 1.0	6.8 ± 0.3
**Total chlorophyll**	12 ± 1.6	12 ± 1.4	15 ± 2.4	12 ± 0.1	16 ± 2.8	11 ± 1.0	16 ± 1.2	13 ± 0.0
**Bioconcentration factor**								
**Y**		115		116		83		44
**Eu**		174		138		75		64

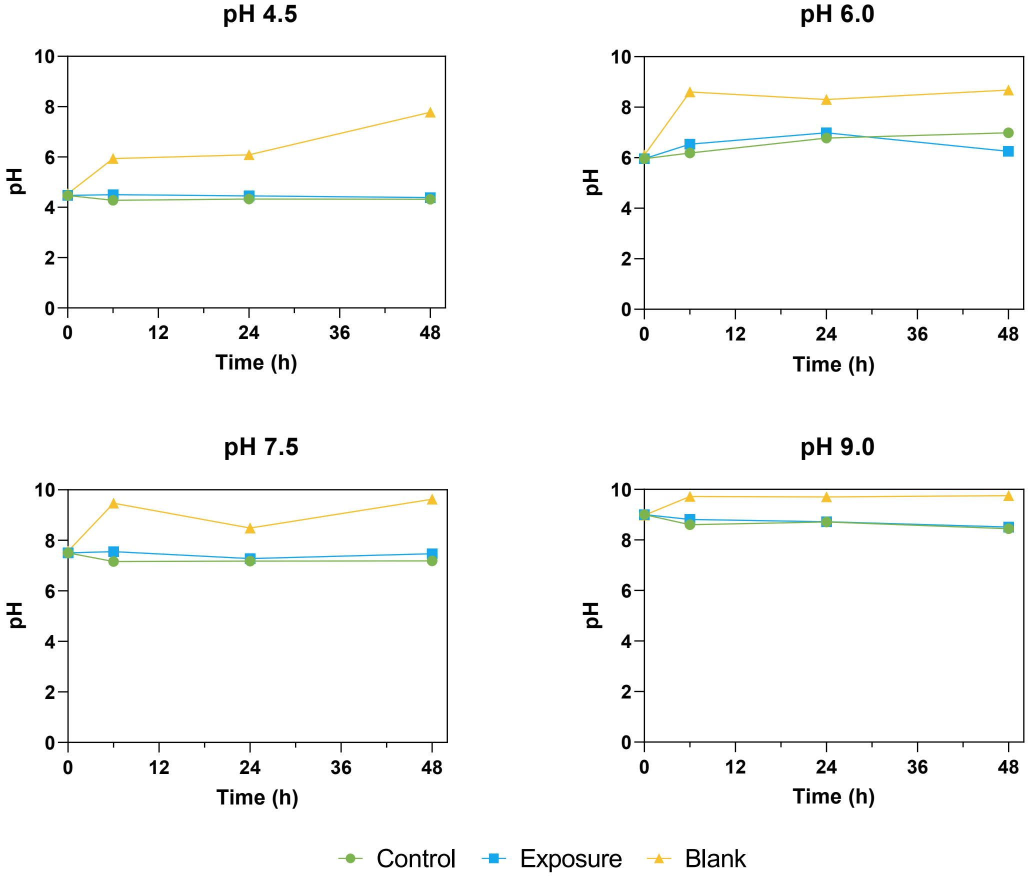

***Figure S1**: Variation of pH versus time of exposure (mean ± standard error).*

***Table S2**: Relative growth rate (%/day), total chlorophyll and bioconcentration factor in the macroalgae exposed to saline water (B) and to the diluted extract (E) at different light exposure (natural light, natural light supplemented with artificial light and absence of light). Chlorophyll analysis was performed with the SPAD-502 meter, thus the units of the analysis are proper of the device.*

	Natural light		Supplemented w/ artificial light		Absence of light	
	B	E	B	E	B	E
**RGR (%/day)**	9.8 ± 2.0	6.5 ± 0.6	7.4 ± 0.6	5.2 ± 0.9	8.4 ± 0.7	5.4 ± 0.3
**Total chlorophyll**	14 ± 1.2	10 ± 1.5	15 ± 1.4	9.6 ± 1.5	12 ± 0.7	10 ± 1.0
**Bioconcentration factor**						
**Y**		203		224		118
**Eu**		155		161		106
**Gd**		162		182		109
**Tb**		187		223		167

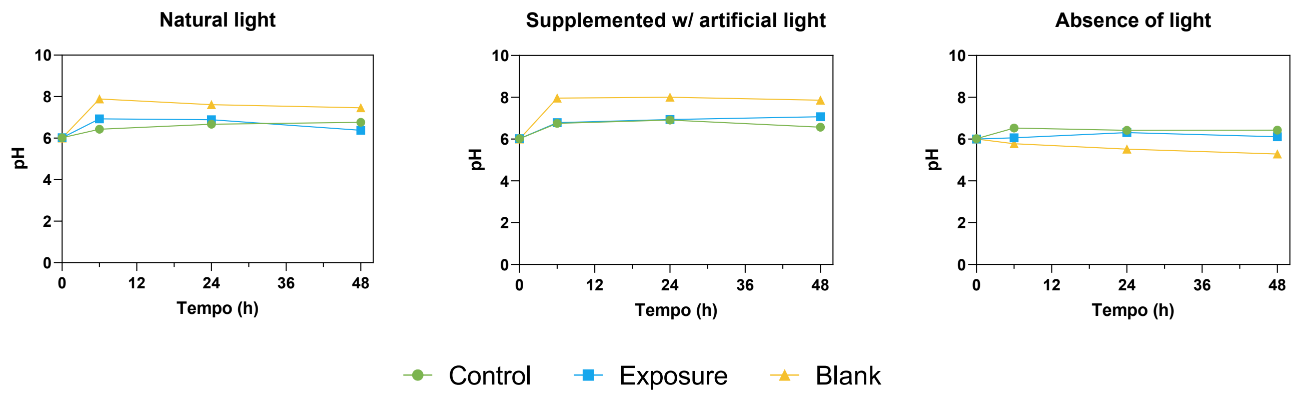

***Figure S2**: Variation of pH versus time of exposure (mean ± standard error).*

***Table S3**: Concentration of REEs and other elements in Ulva sp. biomass ($q_{t\ observed}$, µg/g).*

	Gd	Ce	Tb	La				
**Dried biomass**	45.5	11.9	8.2	< 5.0				
**Calcinated biomass**	323	101	64.1	< 40.0				
	**Al**	**Mn**	**Fe**	**Ni**	**Cu**	**Zn**	**Pb**	**Hg**
**Dried biomass**	1 146	70.8	700	5.6	22.2	16.8	< 10.0	15
**Calcinated biomass**	10 180	568	4 839	-	110	113.7	-	0.3
	**Ca**	**B**	**Mg**	**K**	**Na**			
**Dried biomass**	4 256	70.4	25 988	12 510	31 109			
**Calcinated biomass**	28 302	494	184 314	50 570	88 764			

Table S4: Concentration of REEs and other elements in Ulva sp. biomass ($C_{e\ observed}$, µg/L).

	Gd	Ce	Tb	La
**Solubilized biomass**	366	96.0	66.2	< 40.0

	Al	Mn	Fe	Ni	Cu	Zn	Pb	Hg
**Solubilized biomass**	9223	568	5630	45.3	179	135	< 80.0	4.0

	Ca	B	Mg	K	Na
**Solubilized biomass**	34 268	567	209 110	100 569	250 474

1.2. *Performance comparison with literature values*

Table S5: Application of different adsorbents for REEs recovery. The amount of REEs observed and the maximum adsorbent capacity.

Sorbents	Application to real FL residues	q_t for REE (mg/g)	Langmuir adsorption capacity for REE (mg/g)	References
Al-substituted goethite	no		Eu - 6.8	1
G. sulphuraria (strains SAG 107.79)	no	Y - 5.26; Ce - 9.32; Eu - 20.98 Tb - 20.85	-	2
*G. sulphuraria (*strains ACUF 427)	no	Y - 4.58; Ce - 6.59; Eu - 13.50; Tb - 13.74	-	
ZSM-5 zeolite	no		Eu - 3.3	3
Arthrobacter sp.	no	-	Eu - 9.5	4
Chitosan powder		-	Eu - 48.3	
Chitosan beads		-	Eu - 18.4	
Bioderived Pickering Emulsion Based on Chitosan/Trialkyl Phosphine Oxides	yes	Y - 40	Y - 90	5
Native cellulose	yes	-	Eu - 18.5	6
Saccharomyces cerevisiae		-	Eu - 14.2	
Saccharomyces cerevisiae-immobilized cross-linked cellulose		Eu - 0.08385	Eu - 25.9	
Ulva sp.	yes	Y – 16 – 121; Eu – 0.7 – 5; Gd – 0.045 – 0.32; Ce – 0.012 – 0.10; Tb – 0.0082 – 0.064	-	This study

References

(1) Li, M.; Liu, H.; Chen, T.; Hayat, T.; Alharbi, N. S.; Chen, C. Adsorption of Europium on Al-Substituted Goethite. *J. Mol. Liq.* **2017**, *236*, 445–451. https://doi.org/10.1016/j.molliq.2017.04.046.

(2) Iovinella, M.; Lombardo, F.; Ciniglia, C.; Palmieri, M.; di Cicco, M. R.; Trifuoggi, M.; Race, M.; Manfredi, C.; Lubritto, C.; Fabbricino, M.; De Stefano, M.; Davis, S. J. Bioremoval of Yttrium (III), Cerium (III), Europium (III), and Terbium (III) from Single and Quaternary Aqueous Solutions Using the Extremophile Galdieria Sulphuraria (Galdieriaceae, Rhodophyta). *Plants* **2022**, *11* (10), 1376. https://doi.org/10.3390/plants11101376.

(3) Shao, D. D.; Fan, Q. H.; Li, J. X.; Niu, Z. W.; Wu, W. S.; Chen, Y. X.; Wang, X. K. Removal of Eu(III) from Aqueous Solution Using ZSM-5 Zeolite. *Microporous Mesoporous Mater.* **2009**, *123* (1–3), 1–9. https://doi.org/10.1016/j.micromeso.2009.03.043.

(4) Cadogan, E. I.; Lee, C.-H.; Popuri, S. R. Facile Synthesis of Chitosan Derivatives and Arthrobacter Sp. Biomass for the Removal of Europium(III) Ions from Aqueous Solution through Biosorption. *Int. Biodeterior. Biodegradation* **2015**, *102*, 286–297. https://doi.org/10.1016/j.ibiod.2015.01.018.

(5) Lapo, B.; Pavón, S.; Hoyo, J.; Fortuny, A.; Scapan, P.; Bertau, M.; Sastre, A. M. Bioderived Pickering Emulsion Based on Chitosan/Trialkyl Phosphine Oxides Applied to Selective Recovery of Rare Earth Elements. *ACS Appl. Mater. Interfaces* **2023**, *15* (51), 59731–59745. https://doi.org/10.1021/acsami.3c10233.

(6) Arunraj, B.; Sathvika, T.; Rajesh, V.; Rajesh, N. Cellulose and Saccharomyces Cerevisiae Embark to Recover Europium from Phosphor Powder. *ACS Omega* **2019**, *4* (1), 940–952. https://doi.org/10.1021/ACSOMEGA.8B02845/ASSET/IMAGES/MEDIUM/AO-2018-02845K_M017.GIF.
